# Dichotomous Nitric Oxide–Dependent Post-Translational Modifications of STAT1 Are Associated with Ipilimumab Benefits in Melanoma

**DOI:** 10.3390/cancers15061755

**Published:** 2023-03-14

**Authors:** Saurabh K. Garg, James Sun, Youngchul Kim, Junmin Whiting, Amod Sarnaik, José R. Conejo-Garcia, Mitch Phelps, Jeffrey S. Weber, James J. Mulé, Joseph Markowitz

**Affiliations:** 1 Department of Cutaneous Oncology, Moffitt Cancer Center & Research Institute, Tampa, FL 33612, USA; 2 Department of Surgery, University Hospitals Cleveland Medical Center, Cleveland, OH 44106, USA; 3 Department of Biostatistics and Bioinformatics, Moffitt Cancer Center & Research Institute, Tampa, FL 33612, USA; 4 Department of Oncologic Sciences, University of South Florida, Tampa, FL 33612, USA; 5 Department of Immunology, Moffitt Cancer Center & Research Institute, Tampa, FL 33612, USA; 6 Department of Immunology, Duke University, Durham, NC 27710, USA; 7 Pharmaceutics and Pharmacology, The Ohio State University, Columbus, OH 43210, USA; 8 Perlmutter Cancer Center, New York University Langone Health, New York, NY 10016, USA

**Keywords:** melanoma, ipilimumab, adjuvant therapy, interferon, post-translational modifications

## Abstract

**Simple Summary:**

Nitric oxide is typically thought of as an inhibitory molecule in cancer. In our previous studies, nitric oxide (NO) increased in the immune effector cells among patients with longer RFS after adjuvant ipilimumab, whereas NO increased in the immune suppressor cells among patients with shorter RFS. Herein, we utilize samples derived from the same patients to measure the post-translational modifications of STAT1 (nitration-nSTAT1 and phosphorylation-pSTAT1) important for regulating its activity via flow cytometry and mass spectrometry approaches. Ipilimumab-treated patients with high nSTAT1 levels before and after therapy in PBMCs experienced decreased RFS, but the change in nSTAT1 levels before and after ipilimumab therapy was associated with longer RFS. This study reveals a dichotomous role for nitric oxide in melanoma and may lead to therapeutic implications.

**Abstract:**

Although Ipilimumab (anti-CTLA-4) is FDA-approved for stage III/IV melanoma adjuvant treatment, it is not used clinically in first-line therapy, given the superior relapse-free survival (RFS)/toxicity benefits of anti-PD-1 therapy. However, it is important to understand anti-CTLA-4’s mechanistic contribution to combination anti-PD-1/CTLA-4 therapy and investigate anti-CTLA-4 therapy for BRAF-wild type melanoma cases reresected after previous adjuvant anti-PD-1 therapy. Our group published that nitric oxide (NO) increased within the immune effector cells among patients with longer RFS after adjuvant ipilimumab, whereas NO increased within the immune suppressor cells among patients with shorter RFS. Herein, we measured the post-translational modifications of STAT1 (nitration-nSTAT1 and phosphorylation-pSTAT1) that are important for regulating its activity via flow cytometry and mass spectrometry approaches. PBMCs were analyzed from 35 patients undergoing adjuvant ipilimumab treatment. Shorter RFS was associated with higher pSTAT1 levels before (*p* = 0.007) and after (*p* = 0.036) ipilimumab. Ipilimumab-treated patients with high nSTAT1 levels before and after therapy in PBMCs experienced decreased RFS, but the change in nSTAT1 levels before and after ipilimumab therapy was associated with longer RFS (*p* = 0.01). The measurement of post-translational modifications in STAT1 may distinguish patients with prolonged RFS from ipilimumab and provide mechanistic insight into responses to ipilimumab combination regimens.

## 1. Introduction

The FDA-registered immune-based therapeutics for resected stage III/IV melanoma include anti–PD-1, anti-CTLA-4, and interferon-based therapies. Several clinical trials completed in the past few years have guided clinical care [1,2]. The Eastern Cooperative Oncology Group (ECOG)-1609 (stage IIIB-IV melanoma) trial reported increased relapse-free survival (RFS) with adjuvant anti-CTLA-4 therapies versus high-dose interferon-based therapies (interferon-alfa-2b) [3]. The Checkmate 238 trial compared nivolumab to ipilimumab for the adjuvant treatment of stage IIIB through IV melanoma and demonstrated a 70.5% 12-month RFS rate among the nivolumab group compared to 60.8% among the ipilimumab group, along with significantly decreased toxicity [4,5]. A recently published trial by the Southwest Oncology Group (SWOG) demonstrated increased efficacy of pembrolizumab for stage III melanoma compared to interferon-α or ipilimumab, which were the standards of care at the time [6].

Based on these data, the side-effect profile and the perceived benefit of anti–PD-1–based agents, nivolumab or pembrolizumab are prescribed preferentially in the adjuvant setting for melanoma. Today, ipilimumab is still considered clinically in the setting of a BRAF wild-type patient who is reresected after previous adjuvant anti-PD-1 therapy. Furthermore, the RFS for anti-CTLA-4 regimens at 1 year in various trials ranges from 60% to 70% [3,4]. One of the early trials of adjuvant ipilimumab activity investigated ipilimumab in combination with a peptide vaccine [7]. In 2020, a follow-up study using samples from that trial demonstrated that nitric oxide (NO) had increased in the immune effector cells involved in antigen presentation in patients with longer RFS following ipilimumab, whereas NO had increased in the immune suppressor cells among patients with shorter RFS [8]. Our group reviewed the effects of NO in melanoma, and there are multiple instances where NO mediates immune suppression. In contrast, in other circumstances, NO may promote the ability of immune effector cells to kill melanoma tumors [9,10]. These two papers suggested the dichotomous effect of NO, whereby immune effector cells may utilize NO to kill melanoma cells, whereas immune suppressor cells may use NO to limit the immune effector cells’ ability to kill melanoma cells via the post-translational modification of proteins. This study focuses on the role of STAT1 nitration.

It is known that the presentation of antigens by dendritic cells to T cells is defective in melanoma [11]. Interferon signaling associated with STAT1 phosphorylation at tyrosine 701 by JAKs leads to the dimerization of STAT1 and nuclear translocation that regulates interferon-stimulated genes and leads to the appropriate immune surveillance of cancer [12,13]. The phosphorylation of STAT1 is measured via multiparametric flow cytometry in peripheral blood mononuclear cells (PBMCs) [14]. In contrast to the signal transduction activation by phosphorylation, the nitration of STAT1 in myeloid-derived suppressor cells may cause immune inhibition [15,16].

Our recently published study of patients receiving ipilimumab also demonstrated increased levels of phosphorylated STAT1 (pSTAT1) among patients who had an RFS of more than 12 months, which is consistent with previous studies [10]. Given the overlap in pSTAT1 levels among those patients whose disease relapsed (vs. those whose disease did not), pSTAT1 levels were analyzed herein using Cox regression and Kaplan–Meier analyses. Resting pSTAT1 levels at day 0 and day 150 in adjuvant ipilimumab treatment were compared in addition to the pSTAT1 levels of these same PBMCs after they were stimulated with exogenous interferon-α to illustrate the importance of intermediate levels of interferon stimulation.

The tyrosine located at the 701 amino acid (Y701) of the STAT1 protein can also be nitrated, and the increased nitration of Y701 in both murine splenocytes and human PBMCs is associated with tumor progression [15,16]. In order to investigate STAT1 nitration, our group used selective reaction monitoring (SRM) for the quantitative measurement of STAT1 nitration at Y701, which is the STAT1 phosphorylation site that initiates nuclear translocation [16,17]. The effect of STAT1 nitration in PBMC from patients treated with adjuvant ipilimumab on RFS was examined. The results revealed the importance of time-dependent changes in tyrosine nitration in the PBMCs obtained from patients with melanoma who were undergoing anti-CTLA-4 therapy and experienced long RFS rates. In addition, the data demonstrated the importance of intermediate pSTAT1 levels in PBMCs prior to ipilimumab therapy.

## 2. Materials and Methods

### 2.1. Patient Samples

Archived PBMC samples were obtained from patients who previously participated in a phase II trial of adjuvant ipilimumab with a peptide vaccine, and the treatment regimen has been previously described. [7] Peripheral blood leukocytes were collected before initiation of ipilimumab treatment and at approximately 150 days after the first treatment. PBMCs were available from the previously published study and were de-identified before inclusion in our current study.

### 2.2. Flow Cytometry and Mass Spectrometry Analyses

Flow cytometric analysis of pSTAT1 [14] and liquid chromatography-tandem mass spectrometry (LC-MS-MS) analysis [16] of nitrated STAT1 (nSTAT1) were conducted on all samples. For flow cytometry studies, frozen PBMCs were thawed at 37 °C, washed with culturing media, and allowed to rest overnight in complete media at 5% CO_2_ at 37 °C. PBMCs were stimulated with interferon-α (Miltenyi Biotec, Cambridge, MA, USA) at 0, 10^2^ U/mL, and 10^4^ U/mL and incubated for 15 min in media, as previously described [10].

The live/dead marker Zombie NIR (BioLegend, San Diego, CA, USA) was used before permeabilization to distinguish live cells. The samples were permeabilized using the FIX & PERM Cell Permeabilization Kit with methanol modification (Fisher Scientific, Hampton, MA) and fixed at −20 °C for a minimum of 2 h. pSTAT1 was detected by a pSTAT1 antibody (AF488; BD Biosciences, San Jose, CA, USA). Flow cytometry data were collected either on Canto or LSRII flow cytometers (BD Biosciences, San Jose, CA, USA), and data were analyzed in FCS Express (De Novo Software, Pasadena, CA, USA). Measurement of patient-derived PBMCs for nSTAT1 and native STAT1 via LC-MS-MS SRM experiments was performed as described previously [16].

### 2.3. Statistical Analyses

We conducted statistical analyses to determine whether there is a relationship between levels of pSTAT1, nSTAT1, and RFS. Survival outcome was summarized using Kaplan–Meier method and log-rank testing was performed to evaluate the association of survival outcome with pSTAT1 and nSTAT1 levels dichotomized by the median-split method. The prognostic effect (hazard ratio and 95% confidence interval) of continuous pSTAT1 and nSTAT1 levels, with or without interferon-α stimulation, on RFS was evaluated using a Cox’s proportional hazards regression model. Levene’s F-test for the homogeneity of variance was used to compare the variance of pSTAT1 level among patients with different RFS rates. Patients were also stratified using the median-split method for a survival comparison analysis of [nSTAT1]_post_–[nSTAT1]_pre_ with ipilimumab treatment. The statistical analyses were completed using SAS 9.4, R Studio 3.5.3 (http://www.r-project.org, accessed on 9 March 2023), and GraphPad Prism 7.05. A two-sided *p* value of <0.05 was considered statistically significant. Treatment outcome is reported at the end of the follow-up period for the parent trial (5 years).

## 3. Results

### 3.1. Patient Demographics

Pre- and post-therapy PBMCs were available from 35 patients with resected stages IIIC/IV melanoma. The median patient age was 58 years (range, 21–78 years), and 63% of the patients were male. Sixteen (46%) patients had stage III disease; 19 (54%) had stage IV disease. Six (17%) patients received ipilimumab at 3 mg/kg, and the remaining 29 (83%) received 10 mg/kg. The characteristics of the patient subset used in this study are summarized in Table 1. There were no statistically significant differences in RFS between those patients who received 3 mg/kg and those who received 10 mg/kg (Log rank *p*-value = 0.96).

### 3.2. Type-I Interferon Treatment Increased Phosphorylation of STAT1

It is known that the maximum level of pSTAT1 achievable in PBMCs is different between patients, as described by Lensinski et al. [14]. First, we explored the interferon concentrations needed for the stimulation and saturation of pSTAT1 formation in response to exogenous interferon-α treatment. The normal donor PBMCs were isolated, and we tracked the interferon dose-dependent increase in pSTAT1 levels. Similarly to previous studies [14], an interferon concentration-dependent increase in pSTAT1 levels was seen with increasing interferon levels (Supplementary Figure S1). A concentration of 500 U/mL of interferon-α showed the near-maximum phosphorylation of STAT1 in PBMCs following 15 min of treatment, whereas another normal donor demonstrated increasing pSTAT1 levels throughout the 10^4^ U/mL maximal dose (Supplementary Figure S1). Interferon-α concentrations of 10^2^ U/mL (mimicking subsaturated stimulation) and 10^4^ U/mL (representing *maximum* stimulation of JAK-STAT signaling) were subsequently used for ex vivo stimulation of patient-derived PBMCs.

### 3.3. Phosphorylation of STAT1 Displays a Narrow Distribution in Samples with Long RFS

**Figure 1:**
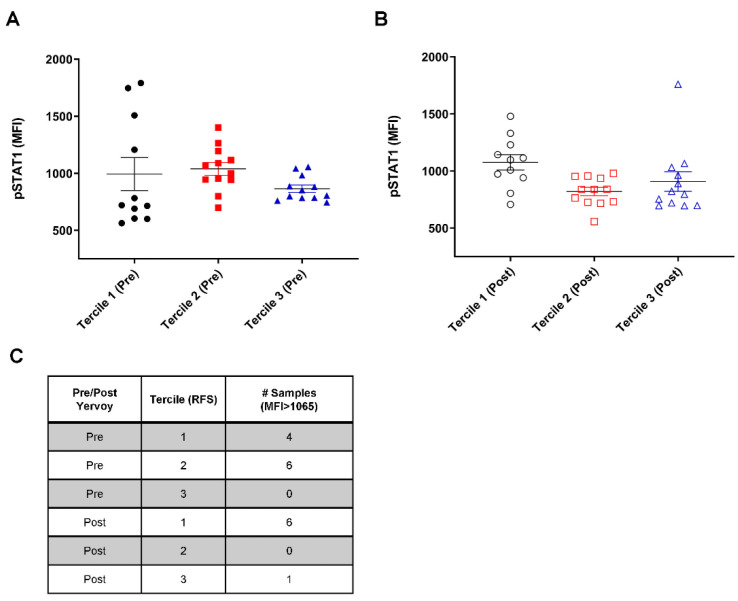
Peripheral blood mononuclear cells (PBMCs) from patients with long relapse-free survival (RFS) display a small variance in levels of phosphorylated STAT1 (pSTAT1). PBMC samples from patients were stratified into RFS terciles before anti-CTLA-4 therapy. Tercile 1 represents the poor outcome, whereas Tercile 3 represents the favorable outcome following anti-CTLA-4 therapy. (**A**) pSTAT1 level measured before ipilimumab administration; (**B**) pSTAT1 level measured after 150 days of adjuvant therapy. Error bar showing ± standard error of the mean (SEM). Abbreviation: MFI, mean fluorescence intensity. (**C**) Table of the number of samples with an MFI greater than 1065.

The relationship between the pSTAT1 levels and RFS was analyzed by dividing the patients into even terciles by RFS. The terciles were defined as follows: tercile 1, RFS < 24 months; tercile 2, RFS between 24 and 40.3 months; tercile 3, RFS > 40.3 months. As shown in Figure 1, the patients in tercile 1 (shortest RFS) had a large variation in the mean pSTAT1 level at baseline before ipilimumab treatment. The patients in tercile 2 (intermediate RFS) demonstrated a less variable distribution in comparison to tercile 1 (*p* = 0.01, Levene’s F-test).

Interestingly, the patients in tercile 3 (longest RFS) demonstrated a narrow distribution of pSTAT1 levels before ipilimumab treatment, and none of the samples displayed a pSTAT1 mean fluorescence intensity >1065, unlike tercile 1 (Figure 1A). This trend of a smaller variance in pSTAT1 among patients with higher RFS rates was retained after ipilimumab administration but did not reach significance, except for comparing tercile 2 to tercile 3 (*p* = 0.008, Levene’s F-test; Figure 1B). These data suggest the importance of intermediate pSTAT1 levels in PBMCs prior to ipilimumab therapy.

### 3.4. Higher Levels of pSTAT1 Were Associated with Low RFS in Anti-CTLA-4 Adjuvant Settings

**Figure 2:**
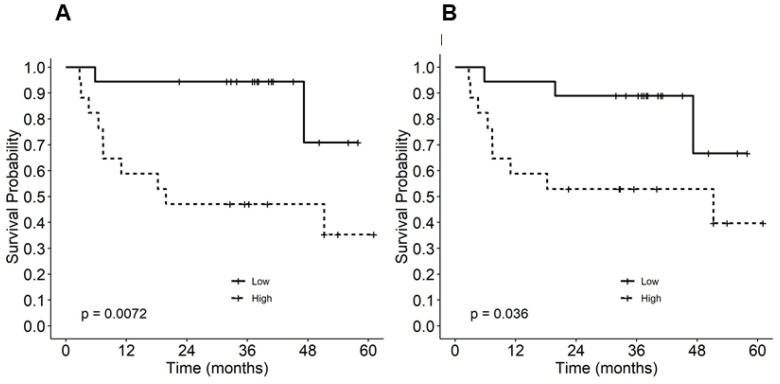
Kaplan–Meier relapse-free survival estimates by phosphorylated STAT1 (pSTAT1) level (**A**) before and (**B**) after ipilimumab treatment.

In order to examine the effect of the steady-state phosphorylation of STAT1 on relapse-free survival in the setting of anti-CTLA-4 adjuvant therapy, we stratified the patients into two groups (above or below the median pSTAT1 level) and analyzed the relapse-free survival probabilities of each group before and after 150 days of ipilimumab administration. The Kaplan–Meier survival curve demonstrated the worst survival in the group with higher pSTAT1 both before (*p* = 0.007; Figure 2A) and after (*p* = 0.036; Figure 2B) adjuvant ipilimumab treatment. Consistently, the patient samples that demonstrated high pSTAT1 levels following stimulation with the exogenous treatment of interferon-α (10^2^ and 10^4^ U/mL) were among the worse survival groups (Supplementary Figure S2). This result suggests that increased pSTAT1 levels beyond a certain point do not lead to favorable outcomes following anti-CTLA-4 adjuvant therapy for melanoma.

### 3.5. Stimulation with 10^2^ U/mL of Interferon-α Demonstrated Comparable pSTAT1 Activation before and after Ipilimumab Treatment

**Figure 3:**
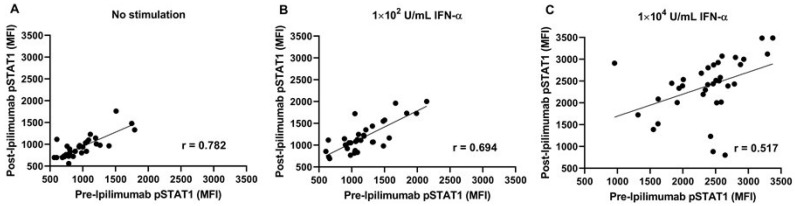
Intermediate interferon-α stimulation resulted in the correlative relationship of phosphorylated STAT1 (pSTAT1) levels pre- vs. post-ipilimumab treatment (**A**) Without interferon-α; (**B**) with interferon-α stimulation, 10^2^ U/mL; and (**C**) with interferon-α stimulation, 10^4^ U/mL.

We studied the effect of anti-CTLA-4 therapy on the capacity of PBMCs to stimulate STAT1 with 10^2^ U/mL and 10^4^ U/mL of interferon-α ex vivo. As is shown in Figure 3A, there is a linear relationship (*r* = 0.78, *p* < 0.001) between pSTAT1 expression on interferon-treated PBMC pre- and post-ipilimumab. There was a linear correlation between the PBMCs treated with intermediate doses of interferon-α (10^2^ U/mL) obtained from patients pre- and post-ipilimumab treatment (*r* = 0.69, *p* < 0.001). This correlation was weakened (*r* = 0.517, *p* = 0.002) at a higher concentration of interferon-α (10^4^ U/mL) (Figure 3B,C). Comparing the correlation coefficients demonstrated no difference between no stimulation and 10^2^ U/mL of interferon-α (*p* = 0.82), whereas a significant difference in the correlation coefficient was found when comparing no stimulation to 10^4^ U/mL of interferon-α (*p* = 0.004) or comparing 10^2^ U/mL of interferon-α to 10^4^ U/mL of interferon-α (*p* = 0.002). The present data suggest that, in post-ipilimumab therapy, PBMCs retain the pretreatment capacity to phosphorylate STAT1 in response to intermediate levels of interferon-α stimulation. However, with a higher dose of interferon-α, this capacity was compromised (Supplementary Figure S3). There were no statistically significant changes in pSTAT1 at 150 days post-treatment between the 10 mg/kg and 3 mg/kg cohorts. Because nitration also occurs at position 701, in the next series of experiments, we investigated the nitration of STAT1 in PBMCs.

### 3.6. Increased [nSTAT1]_post_–[nSTAT1]_pre_ in PBMC Is Associated with Longer RFS

**Figure 4:**
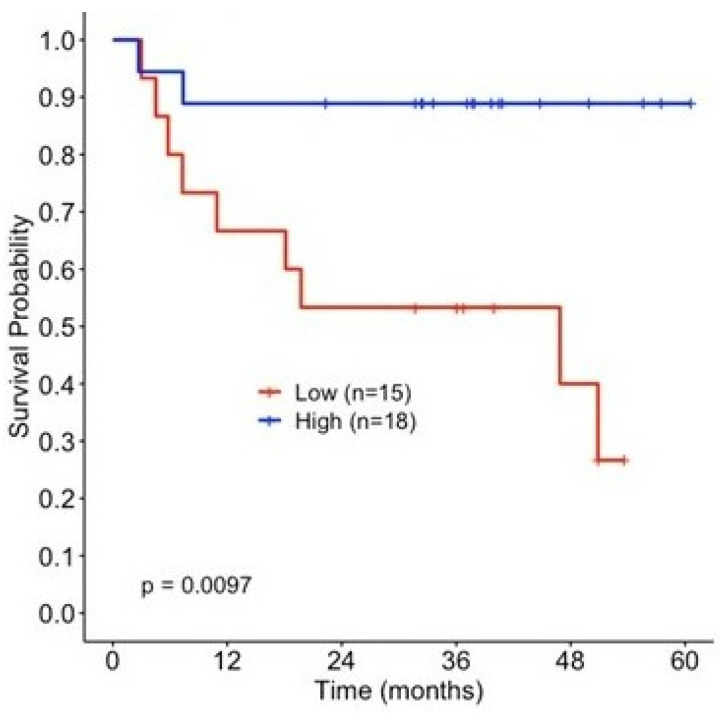
Change in the magnitude of nitration of STAT1 influences relapse-free survival (RFS) following ipilimumab treatment. Kaplan–Meier survival estimates by change in nSTAT1 concentration, stratified by the median (>−0.004, low *n* = 15, high *n* = 18).

Given the above observations for pSTAT1 stimulation pre- and post-ipilimumab therapy, we then analyzed the nitration of Y701 before and after adjuvant therapy at the same time points as were measured for pSTAT1. The magnitude of change in the nSTAT1 levels pre- and post-ipilimumab therapy [nSTAT1]_post_–[nSTAT1]_pre_ was measured via LC-MS-MS SRM techniques. Like the pSTAT1 analyses, the patients were divided into two groups (above and below the median) based on the magnitude of change in the nSTAT1 concentration. Unlike phosphorylation, the nitration of STAT1 did not reveal a significant relationship with RFS either before or after anti-CTLA-4 administration (Supplementary Figure S4). The change in the level of nitrated STAT1 is associated with prolonged RFS. As shown in Figure 4, patients with increased [nSTAT1]_post_–[nSTAT1]_pre_ levels following ipilimumab therapy showed improved relapse-free survival probability, whereas those patients with a lower magnitude of change in nSTAT1 had worse survival (*p* = 0.01). These data also suggest that changes in the nitration of STAT1 tyrosine 701 may influence JAK-STAT signaling after ipilimumab therapy.

## 4. Discussion

A cohort of patients with stage IIIC/IV melanoma was analyzed to examine the relationship between STAT1 post-translational modifications and RFS in response to anti-CTLA-4 adjuvant therapy. A narrow range of stimulation of the interferon pathways, as measured by pSTAT1, is important for the optimal response to anti-CTLA-4 therapy. Furthermore, increases in [nSTAT1]_post_–[nSTAT1]_pre_ correspond to longer RFS among those patients receiving adjuvant ipilimumab. The effects of STAT1 nitration on RFS are dependent on the changes in nitration in STAT1 Y701.

Nitration is a stable post-translational modification [18]. In addition, NO has a dichotomous role, being both immune stimulatory and inhibitory in the PBMCs collected from patients receiving adjuvant ipilimumab therapy [10]. The nitration of STAT1 blocks the phosphorylation of STAT1 and inhibits antigen presentation from dendritic cells to T cells [16]. In other settings, NO may increase the killing of melanoma cells and is reviewed elsewhere [9]. Given the dichotomous effects of NO and the ability of phosphorylated STAT1 to modulate interferon responses, this study measured the nitration of STAT1 in patients who underwent ipilimumab treatment. This is the first report, to our knowledge, of increased survival associated with the nitration of a protein (e.g., STAT1) in melanoma. Nitration inhibits the phosphorylation of STAT1 in vitro and in vivo [15,16]. However, the phosphorylation of tyrosine is more labile than nitration, as suggested by the fact that the nitration of tyrosine at position 701 results in less phosphorylation in murine models [15]. Therefore, limiting the phosphorylation in patients past a certain point may prevent aberrant immune stimulation and subsequent immune suppression and is the next step for investigations in this area. Changes in the nitration status of STAT1 are associated with RFS, which supports the notion that the modulation of the interferon response pathway is important for clinical responses. Interferon-α is FDA-approved and extensively studied for use in melanoma adjuvant therapy, albeit with a high toxicity profile [19,20]. Interferon-α exerts its molecular effects on melanoma in various ways (immunoregulatory, antiangiogenic, and proapoptotic) [21]. It promotes antitumor immunity by enhancing the function of both CD4 and CD8 T cells by positively affecting the maturation, survival, and antigen presentation of dendritic cells [22,23,24]. Indeed, high-dose interferon (HDI) adjuvant therapy of interferon-α (induction therapy consisted of 30 days of 20 MU/m^2^ of intravenous interferon-α daily; maintenance consisted of 10 MU/m^2^ given subcutaneously thrice weekly for one year) was the standard regimen that showed clinical benefits among patients with high-risk melanoma before using more efficacious checkpoint blockade agents [25,26].

In a pilot study of patients treated with HDI, the patients who exhibited changes in pSTAT1 levels after induction around the median had increased RFS, whereas patients who exhibited no change or higher levels of change experienced worse survival rates [27]. Likewise, in our cohort, prolonged RFS was noticed among patients with lower levels of pSTAT1 within a narrow range at baseline (day 0), and similar trends were observed at day 150. Both the pre- and post-therapy data demonstrated that patients with higher levels of pSTAT1 experienced shorter RFS. Likewise, it has been reported that a high dose of interferon-α does not show a more effective induction of interferon-stimulated genes than an intermediate dose [28].

In anti-PD-1 therapy for melanoma, enhanced interferon signaling (before therapy) also results in more favorable outcomes, but markedly elevated or sustained interferon signaling may result in poorer outcomes for patients [8,29,30]. The nitration of STAT1 with adjuvant anti-CTLA4 therapy may modulate interferon signaling to promote longer RFS. These observations provide a foundation for developing future strategies aimed at optimizing interferon signaling in all available adjuvant immunotherapy settings, and these concepts may be expanded to the metastatic setting. Indeed, a pilot study analyzing dose-optimization in HDI settings showed clinical precedence of lowering the standard subcutaneous dose of interferon-α based on the maximal activation (measurement of pSTAT1 levels) of the immune system [31]. The phosphorylation of STAT1 was also utilized as the experimental readout in our study system, given experience and importance.

## 5. Conclusions

In the current study, increased nitration after anti-CTLA-4 therapy is associated with prolonged RFS, whereas, in most cancer studies, increased nitration is associated with immune suppression and disease progression at a single time point. Together with our previous results demonstrating the pro- and antitumor nature of nitric oxide, the results reported herein suggest that the measurement of nitric oxide-dependent events and modulation of interferon-dependent pathways may distinguish patients who have prolonged RFS with anti-CTLA-4 antibodies compared to published responses to anti-PD1 based adjuvant checkpoint blockade in melanoma. The limitations of this study include the sample size, the limited number of patients who have progressed, and the heterogeneity with ipilimumab dosing. The small sample size limits the statistical analysis, as does studying adjuvant-treated melanoma patients with a small number of recurrence events. This study suggests that the nitration of proteins may modulate immune checkpoint blockades, such as ipilimumab, and that it is feasible to measure these modifications from patient samples. Future studies will include patients with metastatic melanoma. In addition, it provides insights into the mechanism of response amenable to the clinical/translational study of combinatory anti-PD-1/CTLA-4 therapy in that moderate interferon responsiveness is best for checkpoint blockade.

## Figures and Tables

**Table 1:** Patient characteristics (*n* = 35).

Characteristic	*n*	Percentage
Gender		
Male	22	63
Female	13	37
Melanoma stage		
IIIC	16	46
IV	19	54
Ipilimumab dose		
3 mg/kg	6	17
10 mg/kg	29	83
Treatment outcome		
NED		77
AWD	2	6
DOD	6	17
Age		-
Median	58 years	
Range	21–78 years	

Abbreviations: AWD, alive with disease; DOD, died of disease; NED, no evidence of disease.

## Data Availability

Data are available upon reasonable request.

## Supplementary Materials

The following supporting information can be downloaded at: https://www.mdpi.com/article/10.3390/cancers15061755/s1, Figure S1: Interferon-α stimulation of normal donor PBMCs. (A) Histogram illustrating pSTAT1 expression with different stimulations of Interferon-α (B) Bar chart of mean fluorescence intensity with different amount of Interferon-α to show the dose-dependent activation of pSTAT1 demonstrating saturation at 10^3^ U/mL (C) Bar chart of mean fluorescence intensity of pSTAT1 with different amounts of Interferon-α illustrating different levels of pSTAT1 stimulation in a second normal donor. Figure S2. Kaplan–Meier survival estimate by pSTAT1 level before (A,B) and after (C,D) ipilimumab treatment. PBMCs were treated with 10^2^ U/mL interferon-α stimulation (A,C) and 10^4^ U/mL Interferon-α stimulation (B,D). Figure S3. Paired pSTAT1 level pre versus post ipilimumab treatment without (A) interferon-α stimulation, after (B) 10^2^ U/mL interferon-α stimulation and (C) 10^4^ U/mL interferon-α stimulation. Although the numbers are small, there is a statistically significant increase (*t*-test, *p* < 0.01) in the levels of STAT1 in the 10 mg/kg cohort, but this does not account for the results seen in this study. Figure S4. Total nSTAT1 levels before and after ipilimumab did not affect RFS. Kaplan–Meier survival estimate by nSTAT1 concentration before (A) and after (B) ipilimumab treatment.
