# Supplementary material for: Dichotomous Nitric Oxide–Dependent Post-Translational Modifications of STAT1 Are Associated with Ipilimumab Benefits in Melanoma
Source: Cancers (Basel). 2023 Mar 14;15(6):1755. doi: 10.3390/cancers15061755 (PMC10046641; doi:10.3390/cancers15061755)
Supplement: Supplementary file 1 [file cancers-15-01755-s001.zip › cancers-2132953-supplementary.pdf]

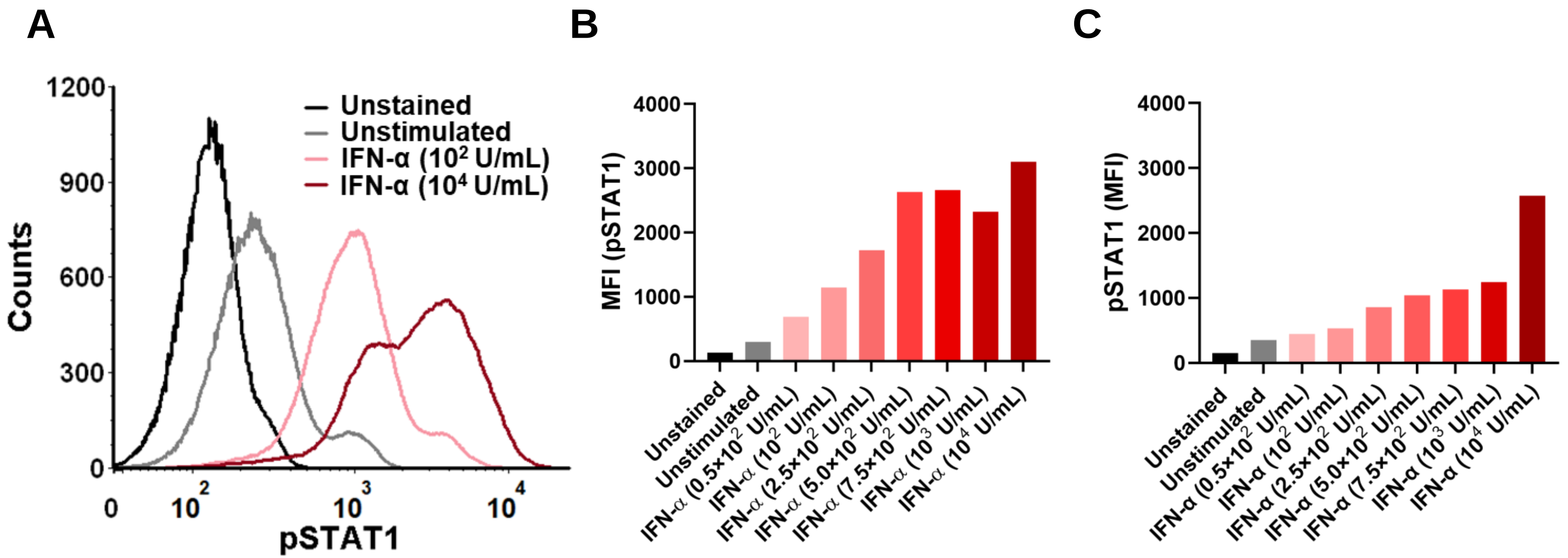

Figure S1. Interferon-α stimulation of normal donor PBMCs. (**A**) Histogram illustrating pSTAT1 expression with different stimulations of Interferon-α (**B**) Bar chart of mean fluorescence intensity with different amount of Interferon-α to show the dose-dependent activation of pSTAT1 demonstrating saturation at 10^3 U/mL (**C**) Bar chart of mean fluorescence intensity of pSTAT1 with different amounts of Interferon-α illustrating different levels of pSTAT1 stimulation in a second normal donor.

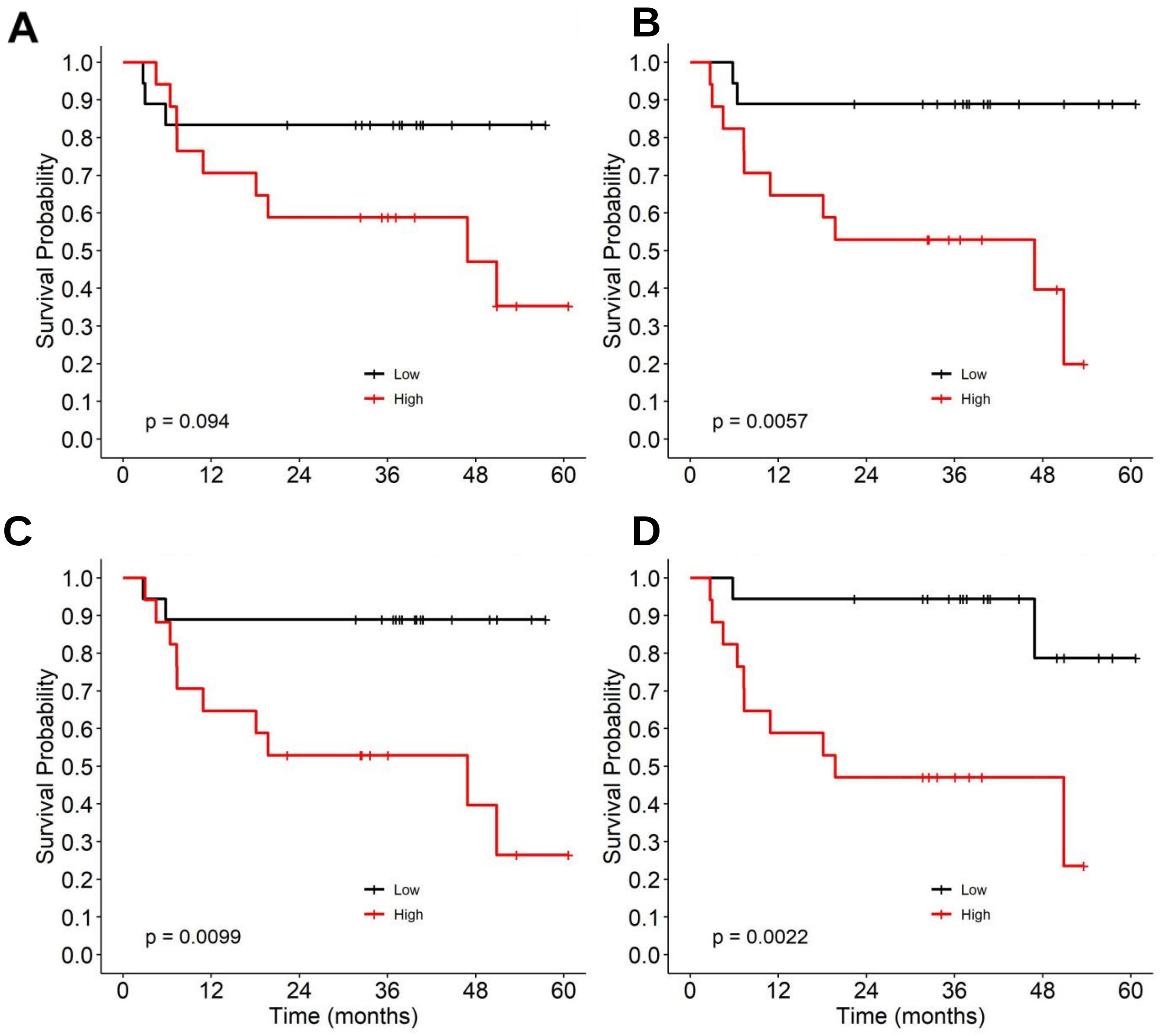

Figure S2. Kaplan–Meier survival estimate by pSTAT1 level before (A,B) and after (**C**,**D**) ipilimumab treatment. PBMCs were treated with 10^2 U/mL interferon-α stimulation (**A**,**C**) and 10^4 U/mL Interferon-α stimulation (**B**,**D**).

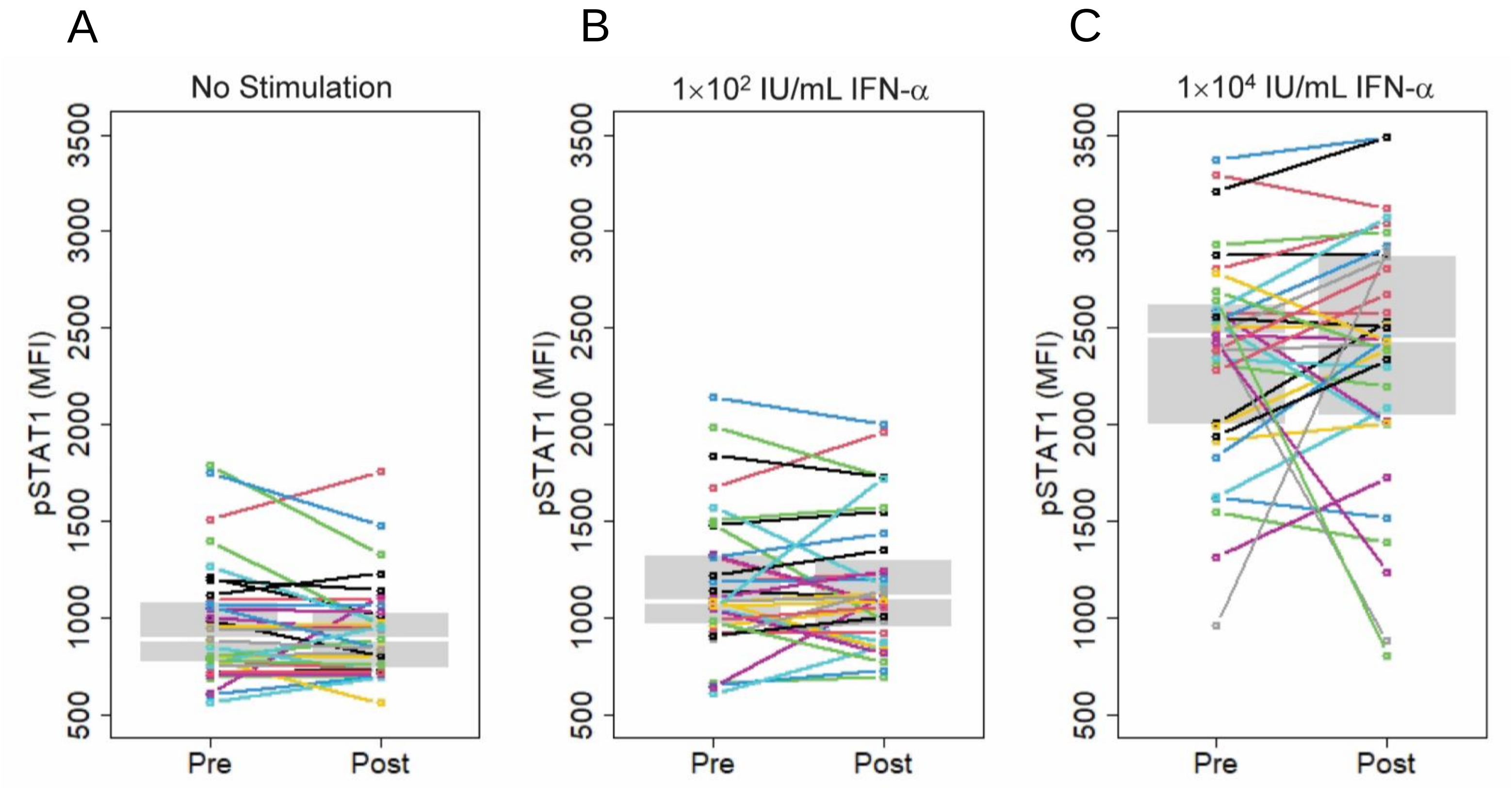

Figure S3. Paired pSTAT1 level pre versus post ipilimumab treatment without (**A**) interferon-α stimulation, after (**B**) 10^2 U/mL interferon-α stimulation and (**C**) 10^4 U/mL interferon-α stimulation. Although the numbers are small, there is a statistically significant increase (*t*-test, $p < 0.01$) in the levels of STAT1 in the 10 mg/kg cohort, but this does not account for the results seen in this study.

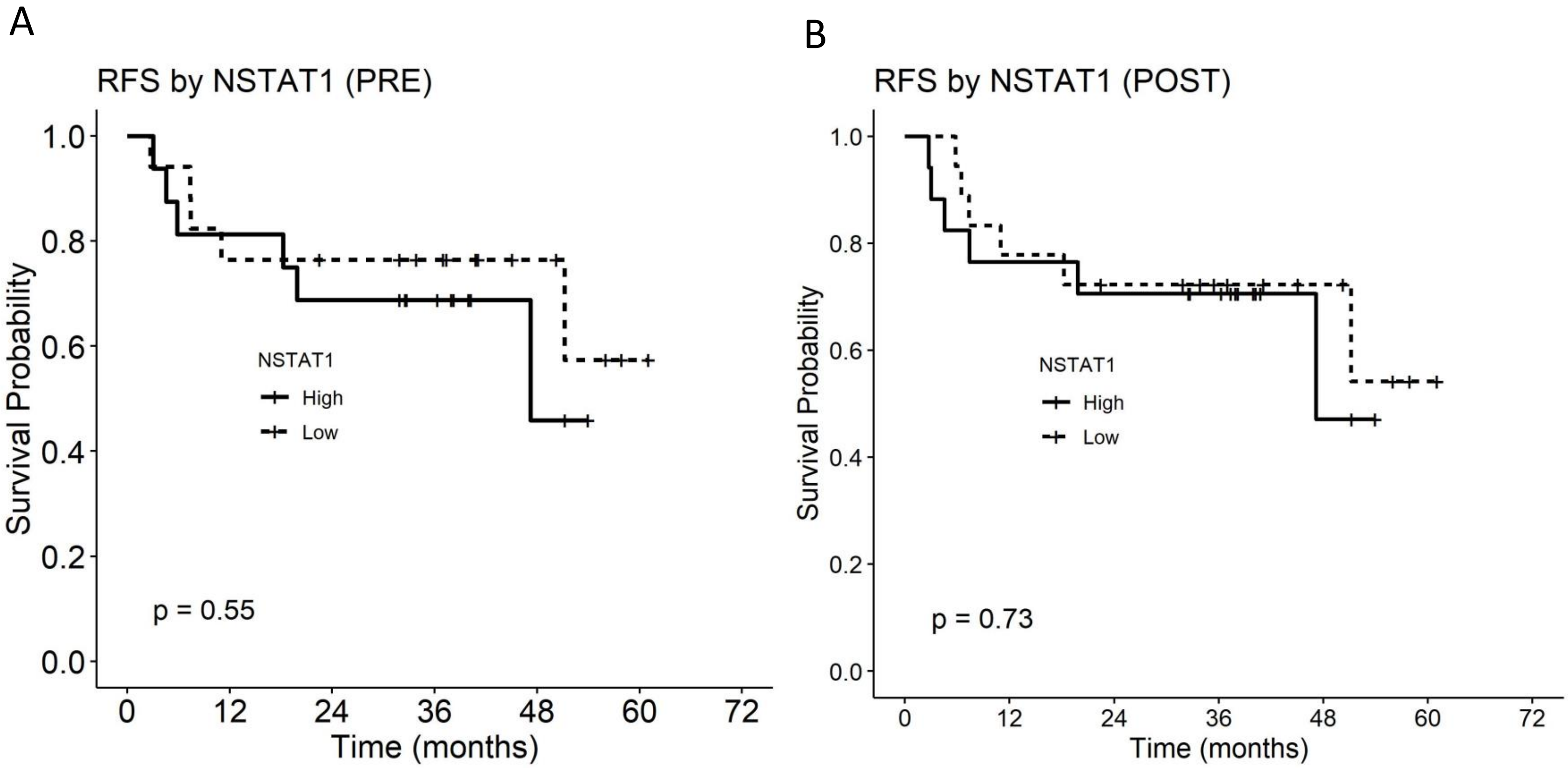

Figure S4. Total nSTAT1 levels before and after ipilimumab did not affect RFS. Kaplan–Meier survival estimate by nSTAT1 concentration before (**A**) and after (**B**) ipilimumab treatment
